# Effect of Glycosylation on an Immunodominant Region in the V1V2 Variable Domain of the HIV-1 Envelope gp120 Protein

**DOI:** 10.1371/journal.pcbi.1005094

**Published:** 2016-10-07

**Authors:** Jianhui Tian, Cesar A. López, Cynthia A. Derdeyn, Morris S. Jones, Abraham Pinter, Bette Korber, S. Gnanakaran

**Affiliations:** 1 Theoretical Biology and Biophysics Group, Los Alamos National Laboratory, Los Alamos, New Mexico, United States of America; 2 Center for Biomolecular Biophysics, Oak Ridge National Laboratory, Oak Ridge, Tennessee, United States of America; 3 Department of Pathology and Laboratory Medicine and Emory Vaccine Center, Emory University, Atlanta, Georgia, United States of America; 4 University of California Berkeley, School of Public Health, Berkeley, California, United States of America; 5 New Jersey Medical School, Rutgers University, Newark, New Jersey, United States of America; National Cancer Institute-Frederick, UNITED STATES

## Abstract

Heavy glycosylation of the envelope (Env) surface subunit, gp120, is a key adaptation of HIV-1; however, the precise effects of glycosylation on the folding, conformation and dynamics of this protein are poorly understood. Here we explore the patterns of HIV-1 Env gp120 glycosylation, and particularly the enrichment in glycosylation sites proximal to the disulfide linkages at the base of the surface-exposed variable domains. To dissect the influence of glycans on the conformation these regions, we focused on an antigenic peptide fragment from a disulfide bridge-bounded region spanning the V1 and V2 hyper-variable domains of HIV-1 gp120. We used replica exchange molecular dynamics (MD) simulations to investigate how glycosylation influences its conformation and stability. Simulations were performed with and without N-linked glycosylation at two sites that are highly conserved across HIV-1 isolates (N156 and N160); both are contacts for recognition by V1V2-targeted broadly neutralizing antibodies against HIV-1. Glycosylation stabilized the pre-existing conformations of this peptide construct, reduced its propensity to adopt other secondary structures, and provided resistance against thermal unfolding. Simulations performed in the context of the Env trimer also indicated that glycosylation reduces flexibility of the V1V2 region, and provided insight into glycan-glycan interactions in this region. These stabilizing effects were influenced by a combination of factors, including the presence of a disulfide bond between the Cysteines at 131 and 157, which increased the formation of beta-strands. Together, these results provide a mechanism for conservation of disulfide linkage proximal glycosylation adjacent to the variable domains of gp120 and begin to explain how this could be exploited to enhance the immunogenicity of those regions. These studies suggest that glycopeptide immunogens can be designed to stabilize the most relevant Env conformations to focus the immune response on key neutralizing epitopes.

## Author Summary

Heavy glycosylation of the envelope surface subunit, gp120, is a key adaptation of HIV-1, however, the precise effects of glycosylation on the folding, conformation and dynamics of this protein are poorly understood. The network of glycans on gp120 is of particular interest with regards to vaccine design, because the glycans both serve as targets for many classes of broadly neutralizing antibodies, and contribute to patterns of immune evasion and escape during HIV-1 infection. In this manuscript, we report on how glycosylation influences an immunogenic but disordered region of gp120. Glycosylation stabilizes the pre-existing conformation, and reduces its propensity to form other secondary structures. It also stabilizes preformed conformation against thermal unfolding. These complementary effects originate from a combination of multiple factors, including the observation that having a glycosylation site adjacent to the disulfide bond further promotes the formation of beta-strand structure in this peptide.

## Introduction

Glycosylation, one of the most common intracellular modifications of proteins[1], is the covalent attachment of one or more carbohydrates (glycans) at specific amino acid sequence motifs. In N-linked glycosylation, the glycan is attached to an asparagine (Asn) residue in an Asn-Xaa-Ser/Thr motif, where Xaa can be any amino acid residue except proline. Based on secondary structure predictions of protein sequences, there appears to be a strong preference for N-linked glycosylation at beta-bends[2], where approximately 70% of N-linked glycan motifs occur, while 10% and 20% occur in alpha-helices and beta-sheets, respectively[1]. Lentiviral envelope proteins are among the most heavily glycosylated proteins in nature[3]. Carbohydrates constitute half of the HIV-1 Env gp120 mass, and cover much of its surface[4].

It has long been known that gp120 can accommodate a remarkable heterogeneity in terms of the number and location of glycosylation sites [5]. This variably glycosylated protein mediates the interactions with CD4 and coreceptor molecules that are critical for viral entry. However, the effects of glycosylation on the conformation and biology of gp120 are not well understood. In general, glycosylation can stabilize protein conformation[6], accelerate protein folding[7], promote secondary structure formation[8], reduce protein aggregation [6, 9], shield hydrophobic surfaces[10], promote disulfide pairing[11], and increase folding cooperativity[12]. Others have shown that glycosylation can stabilize a protein structure against thermal unfolding due to entropic effects[13, 14]. In some cases, glycosylation can slow down the folding process by stabilizing the on-pathway folding intermediates[15]. These varied effects of glycosylation on protein stability are sensitive to the number and location of glycans in the tertiary protein structure[16–20]. Furthermore, modeling approaches typically neglect the influence of non-specific and specific protein-protein and protein-glycan interactions, which play an important role in glycosylation effects[19, 21–25]. Despite recent computational studies of glycosylation[14, 23, 26–28], the effects of carbohydrate moieties on protein conformation and folding are incompletely understood, particularly when glycosylation occurs in or near a region with an unstructured conformation.

HIV-1 gp120 contains multiple highly immunogenic regions and serves as the major target for neutralizing antibodies. The network of glycans on gp120 is of particular interest with regards to HIV-1 vaccine design, because the glycans both serve as targets for many classes of broadly neutralizing antibodies[29–32], and contribute to patterns of immune evasion and escape during HIV-1 infection[33–39]. Elucidating the relevant forms of glycans for neutralizing antibody epitope formation could aid in the design of glycopeptide-based vaccine immunogens for HIV (for examples, see: [33, 40–42]).

**Fig 1:**
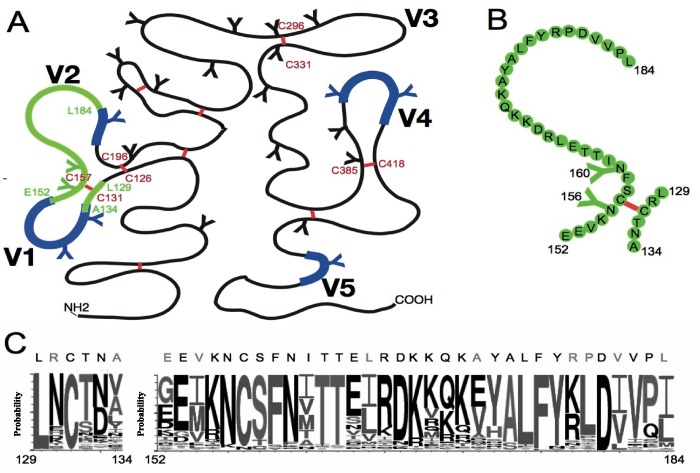
Map of gp120 variable domains and diagram of the V1V2 peptide construct considered in this study. (**A**) Cartoon diagram showing the variable regions of gp120 (V1 –V5), disfulfide bonds, and structural region corresponding to the peptide construct in the context of gp120. Blue–hyper-variable regions; Red—disulfide bonds; Green–V1V2 peptide region. This cartoon is modified from the original Leonard reference [49]. (**B**) The peptide construct (green) contains 6 amino acids from V1 and 33 amino acids from V2 connected by a disulfide bond (red). The sequence corresponds to the CAP45 strain **(C)** The sequence variability of the regions encompassing the peptide construct among HIV-1 isolates shown as a web logo (http://www.hiv.lanl.gov/content/sequence/ANALYZEALIGN/analyze_align.html). All residues are numbered according to the HXB2 reference sequence.

In this study, we investigated glycosylation patterns in the gp120 variable domains using an updated set of 4633 HIV-1 sequences from the 2014 Los Alamos HIV Database reference alignment. Our analysis highlights the enrichment of glycosylation at the base of the 4 disulfide bonded variable loops in gp120 (**Fig 1A**). This led us to focus on the effects of glycosylation at two conserved sites in the V1V2 domain that are proximal to the Cys at 157: the site at 156 which is immediately adjacent, and one at 160 that is nearby (**Fig 1B**). This region constitutes an important glycan-dependent target of broadly neutralizing antibodies (**Fig 1B**) [29–31, 43, 44]. This region of V1V2 contains both conserved and highly variable positions as captured in **Fig 1C**. It tends to maintain a region of high positive charge, and appears to have a flexible conformation, exhibiting a tendency to adopt different conformations. In the native trimer form of HIV-1, this region of V1V2 adopts a beta-strand conformation, which persists when it is bound to broadly neutralizing antibodies PG9 and PG16 [30, 44–46]. However, the V1V2 region is found in several conformations such as beta-strand, helical and random coil when bound to monoclonal antibodies (mAbs) elicited by RV144 vaccination that have limited neutralization breadth [45, 47]. These findings suggest that certain conformations of V1V2 could preferentially elicit antibodies with neutralization breadth, and several groups are actively working towards this goal by developing V1V2-derived glycopeptide immunogens [40, 48]. However, the conformational variability of this region, further complicated by glycosylation, could present substantial obstacles to this strategy.

**Fig 2:**
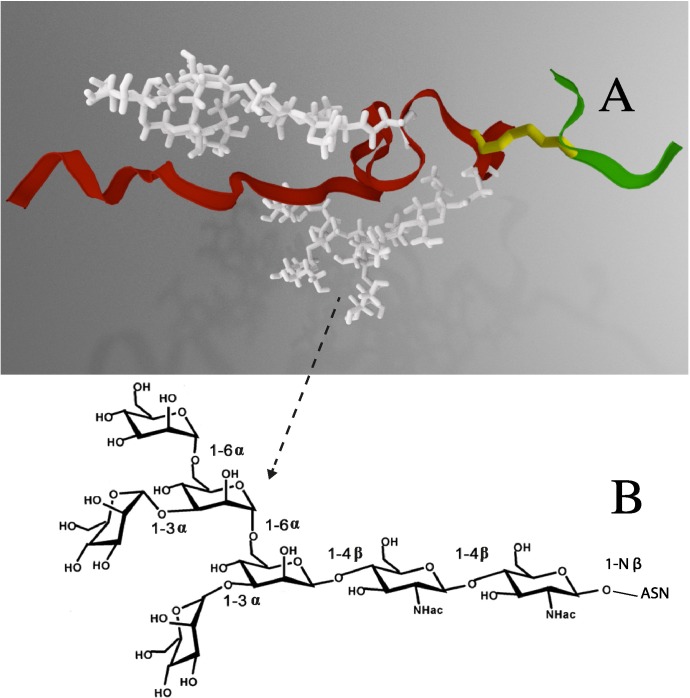
A three-dimensional image of the glycosylated peptide construct from the CAP45 strain. **(A)** A ribbon diagram shows the gp120 V1 fragment in green, and the V2 fragment in red. A disulfide bond is shown in yellow, and the carbohydrates corresponding to sites N156 and N160 are shown as sticks in white **(B)** The chemical composition of high-mannose glycan, (Man)_5_(GlcNAc)_2_, is shown.

To better understand how glycosylation influences this immunogenic but disordered region of V1V2, we utilized an unbiased all atom MD simulations approach. A peptide construct (**Fig 1B**) was generated to mimic this immunologically important region of V1V2; 6 amino acids from V1 (129–134 HXB2 numbering) and 33 amino acids (152–184 HXB2 numbering) from V1 and V2 (including the region that was targeted by vaccinees in the RV144 trial and by other V2-directed antibodies from chronic HIV-1 infection), connected by a disulfide bond between the two cysteine residues Cys 131 and Cys 157 that close the V1 loop (**Figs 1B and 2A**). The folding of this peptide, with and without the carbohydrates at positions N156 and N160, was studied. We show that glycosylation has numerous effects on the stability and accessibility of this disulfide linked V1V2 peptide that could influence the antigenicity and immunogenicity of this region.

## Methods

The all-atom replica exchange molecular dynamics (REMD) simulations were used to analyze conformational aspects of a peptide construct derived from the V1V2 variable loop region of HIV-1 gp120. The peptide construct contains 6 amino acids from V1 (HXB2 gp120 residues 129–134) and 33 amino acids from V2 (HXB2 gp120 residues 157–184) connected by a disulfide bond between Cys131 in V1 loop and Cys157 in V2 loop as shown in **Fig 2A**. The sequence of the peptide corresponds to that of the CAP45 strain, (Genbank accession number GQ999974) it was selected because a PG9 bound structure using CAP45 is available [30]. Our model included two glycans attached to the peptide at positions N156 and N160, which, as noted, have been shown to be important for recognition by several broadly neutralizing antibodies. Also, N156 is immediately adjacent to the disulfide bridge formed by cysteines 131 and 157. This specific sequence did not contain a glycan at position 130 that is found in many HIV-1 strains (**Fig 1C**). Several studies using virion-associated envelope or SOSIP trimer proteins have shown that the carbohydrate moieties of HIV-1 virions are almost entirely oligomannose, consisting mainly of varying proportions of Man_5-9_GlcNAc_2_. However, Man_5_GlcNAc_2_ is consistently present at key glycan positions in gp120, including N156 and N160 [50–53]. Given the consistent presence of Man_5_GlcNAc_2_, in relevant forms of HIV-1 envelope, and that this biosynthetic intermediate reflects the inherently restricted glycan processing of HIV-1 envelope, the carbohydrate moieties used in our model are high-mannose Man_5_GlcNAc_2_ (**Fig 2B**). Furthermore, molecular dynamics simulations of the BG505 SOSIP trimer with Man_5_GlcNAc_2_, Man_7_GlcNAc_2_, or Man_9_GlcNAc_2_ at every sequon produced concordant results in terms of overlap with volume occupied by broadly neutralizing antibodies [53]. Separate REMD simulations were carried out for the unglycosylated and glycosylated peptide construct to quantitate the effects of glycosylation. Additionally, we carried out REMD simulations on a single peptide fragment (NCSFNVTTIVRDKTTK) derived from the HIV-1 subtype C consensus sequence (ConC; Los Alamos Database Consensus Alignments, 2013) with or without glycosylation at N160, and in the absence of a disulfide bond. These simulations were used to independently verify the glycosylation effects seen in the primary simulation studies on the peptide construct.

All systems were composed of peptide/glycopeptide, water, and counter-ions. A total of 84 replicas were performed for the primary simulations, with a temperature range covering 275 K to 550 K. Each replica was run for 500 ns. A total of 76 replicas were included in the second set of simulations, with the temperature range covering 285 K to 558 K. Each replica was run for 100 ns. The details of the four REMD simulations are shown in **Table 1.** The second halves of the REMD trajectories were used for analysis.

**Table 1:** Systems studied by MD simulations.

System	Strain	No. of Glycans	Disulfide	Waters	No. of Atoms	Method	No. of Replicas	Time, ns (replica)
V2	CAP45	0	Yes	8459	26057	REMD^a^	84	500
V2g	CAP45	2	Yes	8240	25718	REMD	84	500
V2_c	ConC	0	No	5842	17790	REMD	76	100
V2g_c	ConC	1	No	5821	17844	REMD	76	100
V2_h	CAP45	0	Yes	6944	21413	MD^b^	20	60
V2g_h	CAP45	2	Yes	7383	23051	MD	20	100

a. REMD, replica exchange molecular dynamics simulations

b. MD, conventional molecular dynamics simulations

The AMBERff99SB[54] force field parameters were used for the peptide, and GLYCAM06[55] force fields were used for the carbohydrate moiety, as they have been shown to be compatible with each other for glycoprotein studies[55]. Constant temperature and constant volume (NVT) REMD simulations were conducted to sample the conformational space [56], REMD is an enhanced sampling technique based on the parallel tempering Monte Carlo method [56–59], where copies of identical systems are simulated at different temperatures. Periodically state exchange between replicas is attempted, and the acceptance rule for each move between states i and j, dictated by a Boltzmann distribution, is $P_{acc}=min\left\{1,exp[(\beta_{i}-\beta_{j})(U({\vec{r}}_{i}^{N})-U({\vec{r}}_{j}^{N}))]\right\}$, where *β = 1/k*_*B*_*T* and $U({\vec{r}}_{i}^{N})$ represents the configurational energy of the system in state i. Together with the exchange, the particle momenta are scaled by *(T*_*i*_*/T*_*j*_*)*^*1/2*^, such that the kinetic energy terms in the Boltzmann factor cancel out[56]. REMD sampling can also be described in terms of umbrella sampling[60]. The temperature spacing between replicas was chosen to ensure sufficient energy distribution overlap between neighboring replicas such that exchange attempts were, on average, accepted with a 20% probability[61]. The potential energy distributions of the system were simulated at constant volume and constant temperature for 30 different temperatures to establish the distribution for the replicas[62].

In addition, thermal unfolding studies of the peptide construct were carried out to characterize the effects of glycosylation on unfolding. Conventional MD simulations at high temperature were conducted on the fragment of the V1/V2 variable loop starting from the beta-strand structure (PDB ID: 3U4E). Two sets of simulations were done, with each set having 20 replicas: one set was for peptide with two glycans at N156 and N160, and the other set was for peptide without glycans. All 20 replicas in each set started from the same conformation but ran with different random seeds at 450 K. Simulations of 60 ns were run for each one without glycan to see the peptide completely unfold, while simulations of 100 ns were run for each one with glycan, and significant amount of beta-strand remained at the end of simulations. The details of the systems are listed in **Table 1**.

The Nose Hoover thermostat was used for the temperature coupling with a coupling time constant τ_T_ = 1.0 ps. The protein/glycoprotein and solvent are coupled separately to thermostats with the same coupling parameters. Van der Waals interactions are treated using a 1.0 nm cutoff. The electrostatic interactions are treated by smooth particle mesh Ewald summation. All bond interactions involving hydrogen atoms are constrained using SETTLE and SHAKE to allow a 2 fs integration time step. A total of 102.4 μs simulations have been carried out in the current study.

Configurational entropy calculations were performed following the formulation of Schlitter[63]. This approach provides an approximate value (upper bound) *S* to the true configurational entropy *S*_true_ of the simulated system,

$$S_{true}<S=\frac{k_{B}}{2}\ln\;det[1+\frac{k_{b}Te^{2}}{\hbar^{2}}\underline{D}]$$

where *k*_B_ is the Botlzmann’s constant, T is the absolute temperature, e is Euler’s number, and ℏ is Planck’s constant divided by 2 π. Here $\underline{D}$ is the covariance matrix of mass-weighted atomic Cartesian coordinates, defined as

D_=〈[M_12(r−〈r〉)]⊗[M_12(r−〈r〉)]〉

where **r** is the 3N-dimensional Cartesian coordinate vector of N particles (atoms or beads) considered for the entropy calculation after least-squares fitting onto a referenced structure, $\underline{M}$ is the 3N-dimensional diagonal matrix containing the masses of these particles, <…> denotes ensemble averaging, and the notation **a** ⊗ **b** stands for the matrix with elements *μ*, *ν* equal to **a**_*μ*_ * **b**_*ν*_. In our case, the structures after minimization were used as reference for the least square fitting of 1200 snapshots of the trajectory. Moreover, during the fitting procedure, the rotational and translational contribution was removed, considering only the internal degrees of freedom of the peptide backbone.

Finally, we carried out large-scale all-atom MD simulations of the glycosylated and the unglycosylated Env spike containing gp120 trimers [64]. Initial coordinates of the glycoprotein complex were downloaded from the protein data bank repository (PDB code 4NCO) [65]. Missing loops and residues in the structure were built using the Modeller package [66] using the full sequence of the BG505 SOSIP gp140 Env trimer in complex with broadly neutralizing antibody PGT122 [65]. The X-ray structure is partially glycosylated with mannose sugar derivatives and all glycosylation positions were completed to have the same Man_5_ carbohydrate moieties. The system is represented using the same force field as used in the simulations of the V1V2 peptide fragment (described above). Env trimeric spike was placed in a cubic box of ~ 4000 nm^3^ and was solvated with 100000 water molecules.

Two independent MD simulations were carried out for the Env spike with and without glycosylation. Each simulation was run for one microsecond and carried on using the GROMACS 4.6.5 [67] molecular simulation package. All atom simulations were performed using a 2 fs time step to integrate Newton’s equations of motion. The LINCS algorithm [68] was applied to constrain all bond lengths with a relative geometric tolerance of 10^−4^. Non-bonded interactions were handled using a twin-range cutoff scheme. Within a short-range cutoff of 0.9 nm, the interactions were evaluated every time step based on a pair list recalculated every five-time steps. The intermediate-range interactions up to a long-range cutoff radius of 1.4 nm were evaluated simultaneously with each pair list update and were assumed constant in between. A PME approach [69] was used to account for electrostatic interactions with a grid spacing set to 0.15 nm. Constant temperature (300 K) was maintained by weak coupling of the solvent and solute separately to a Berendsen heat bath [70] with a relaxation time of 1.0 ps. Similarly, an isotropic approach was used to couple the pressure of the system to 1.0 bar. Trajectories were stored every 20 ps for further analysis.

## Results

### Glycosylation patterns in the gp120 hyper-variable domains

HIV-1 gp120 contains between 18 to 33 N-linked glycosylation motifs, and maintains a median of about 25 despite very high levels of genetic variability[71]. Some of the glycan sites are highly conserved across all of the major HIV-1 clades and circulating recombinant forms, while a subset show clade-specific patterns[71, 72]. However, considerable diversity in gp120 amino acid sequence and glycosylation is evident even in single individuals over time[71], and this variation can mediate antibody immune escape[34–39, 73, 74], including cases where the carbohydrate is a direct component of the epitope [30]. The range in number of glycan sites is largely a consequence of mutations, insertions and deletions that occur within the four hyper-variable domains of gp120 (V1, V2, V4 and V5) (see **Fig 1A**), with insertions often reflecting local imperfect direct repeats of varying lengths[75]. The V3 domain is also variable, however, it is more conserved than the four hyper-variable regions in terms of length and variation of glycosylation sites [71].

**Fig 3:**
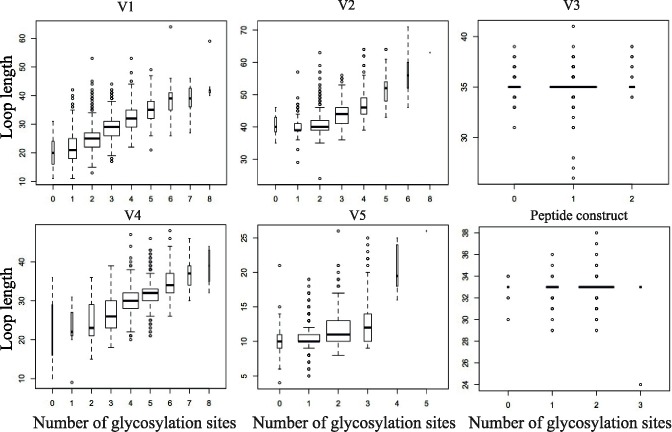
The relationship between loop lengths and number of glycosylation sites in the gp120 variable regions V1-V5. These plots are based on the Los Alamos Database (www.hiv.lanl.gov) alignment, which contains 4,633 curated HIV-1 Env sequences. This alignment contains intact, full length Env sequences, and includes only one sequence per sampled individual. Sequences of poor quality (frameshifts, ambiguity codes or inappropriate stop codons) were excluded from the alignment. The relative width of the box plots is proportional to the square root of the number of sequences in this set that have a given number of potential N-linked glycosylation sites, having the sequence pattern (NX[ST]), were N is an Asparagine, followed by X, any amino acid except Proline, followed by either a Serine or Threonine. Also shown is the epitope region, spanning HXB2 positions 152–184, from the V1V2 peptide construct. In the case of hyper-variable loops, V1, V2, V4, and V5, a p-value < 2.2e-16 was estimated for the correlation between length and number of N-linked glycosylation sites using Kendall's *tau* statistic (estimated using the R statistical package; it is non-exact due to ties and large sample sizes). The Variable Length Characteristics tool was used to evaluate these regions, and the full loop regions were included in the analysis (V1, HXB2 positions 131–157; V2 158–196; V3 296–331; V4 385–418; and V5 360–469).

**Fig 4:**
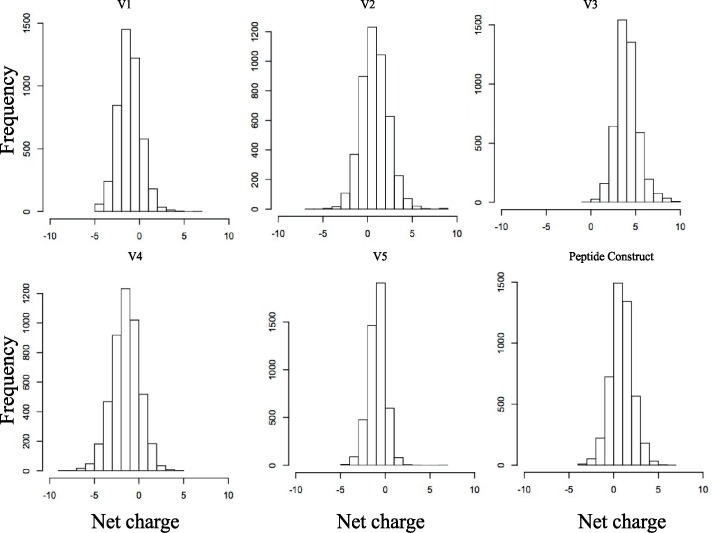
Net charge distribution of the variable regions. Using the same input data as in Fig 3, charge variability in the V-1V5 regions and the V2 epitope region is shown. The V3 loop and V2 epitope region, despite being conserved in terms of length and number of glycosylation sites (**Fig 3**), both show a great deal of variation in net charge, comparable to the level of diversity found in hyper-variable regions. Net charge is calculated as the sum of positive and negative charges, where amino acid residues E and D are assigned -1, and K, R, and H are assigned +1. The V2 epitope region, V3 and V2 tend to be positively charged; V1, V4, V5 tend to be negatively charged.

To investigate patterns of glycosylation in gp120, we first performed an updated analysis of the relationship between hyper-variable loop length and glycosylation in gp120. We included the most current set of global sequence data (n = 4,633) and used a bioinformatics tool that is newly available at the Los Alamos HIV database (http://www.hiv.lanl.gov/). **Fig 3** illustrates that for the four loops that are hyper-variable in terms of insertions and deletions (V1, V2, V4, and V5), the loop length and number of glycan sites are highly variable and correlated with each other; in contrast these parameters for the V3 region are almost invariant. The V2-epitope region we have used as a basis for the peptide we model here is similar to V3 in that it is almost invariant in terms of length and number of potential glycosylation sites; the hyper-variable regions that evolve by insertion and deletion in the V1 and V2 loop flank the key epitope region represented in the peptide, in the context of the natural protein (In **Fig 1A**, the hyper-variable regions within the loops are indicated in blue). In contrast, there is broad net charge distribution in all of these variable regions (**Fig 4**), including the epitope region, which varies from -4 to +7 for V1, 2, 4 and 5, and ranges from -1 to +10 for V3. Whereas V3 has a positive charge, with a median of +4, the median charge is close to neutral for the other variable domains [76, 77].

### Cysteine-proximal glycosylation sites are preferentially found at the base of the hyper-variable loops in gp120

**Fig 5:**
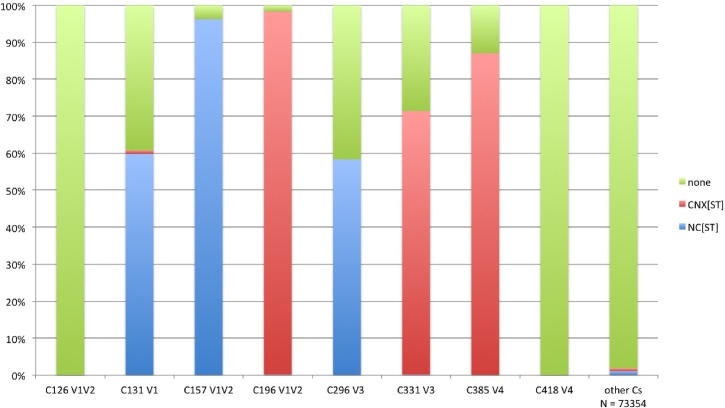
Six out of the eight conserved cysteines located at the base of HIV-1 variable loops V1-V4 are very frequently proximal to a N-linked glycosylation site. Of the 4633 sequences in the filtered alignment in the 2014 database, there are on average 24 Cys per gp160, including 8 that close the variable loops V1-V4 and 16 others. Among the 8 conserved Cys that form disulfide bonds at the base of V1-V4, 21,837 immediately neighbor an N-linked glycosylation site, or 59%. These are concentrated in positions Cys131, Cys157, Cys196, Cys296, Cys331, and Cys385. Among the 73,354 Cys that are not located at the base of the variable loops, proximal glycans are very rare at 1.8%. Of the 6 Cys with conserved proximal glycans in HIV, only the most conserved, Cys157 and Cys196 are also highly conserved in 14 SIVCPZ sequences.

The V1, V2, V3, and V4 loops are each delineated by an invariant cysteine-cysteine disulfide bond, and for 6 of these 8 loop-bounding cysteines, N-linked glycosylation sites tend to be immediately adjacent to one or both of the cysteines. There are two possible sequence motifs for these patterns: NCS/T or CNXS/T, where X can be any amino acid except for proline (**Fig 1A and S1 Fig**). Among the 4633 sequences analyzed, there are 8 conserved Cys residues at the bases of the variable loops, and on average, another 16 Cys residues per Env. Outside of the variable loops, these other Cys residues rarely have a proximal N-linked glycosylation site (**Fig 5**; 1357 of 73354, or 1.8%). In contrast, the majority of the conserved Cys at the bases of the variable loops are proximal to a glycan (**Fig 5**; 21,837 out of 37,064, 59%). The enrichment for Cys-proximal N-linked glycosylation at the bases of the hyper-variable loops is highly significant (6/8 vs 0/16, p = 0.0002). In particular the two N-linked glycosylation sites (N156, N197) that are proximal to the Cys residues at the base of the V2 loop (corresponding to C157, C196: **Fig 5**) are both very highly conserved across HIV-1 subtypes (S2 **Fig**). This suggests that these Cys-proximal glycans are important for Env function, and may impact the structural conformation of the variable loops in the intact protein, and V2 in particular.

### V1V2 peptide construct containing both glycosylation and disulfide linkage spans a critical immunodominant region

A beta-strand of 33 amino acids that contains key neutralizing antibody epitopes is embedded in our peptide construct (**Fig 1A**). The portion that resides within V2 is conserved in terms of length and number of glycosylation sites; 94% of the sequences are 33 amino acids long in this region, and 88% carry 2 glycosylation sites (generally the highly conserved sites at N156 and N160), while 11% have only one (S2 **Fig**). Despite overall conservation in length and number of glycans (**Fig 3** and **S3 Fig**), this V2 ‘epitope’ region has many highly variable positions (**Fig 1C**), and it can vary dramatically in net charge (**Fig 4** and **S3 Fig)**. Nevertheless, this region is of great interest from a vaccine perspective because it serves as the contact region for glycan-dependent broadly neutralizing antibodies such as PG9 and PG16 that recognize the V2 region with a preference for the quaternary structure [29, 30]. This region is also recognized by other broadly neutralizing antibodies, PGT141-PGT145 [31], CH01-CH04 [32] and the VRC26 group of mAbs [43] as well as by a number of antibodies with narrower neutralization breadth, such as C108g [78], 10/76b [79] and 2909 [80] isolated from infected or immunized animals. The two conserved N-linked glycans that are in this region form parts of these epitopes and directly contact antibodies PG9 and PG16 [29, 30]. In addition, in the RV144 vaccine trial immune responses to this linear epitope region were correlated with reduced risk of infection, and the protective effect was associated with antibody-dependent cellular cytotoxicity (ADCC), not neutralization [81, 82].

### Glycosylation reduces the propensity of the V1V2 peptide to adopt folded secondary structures

**Fig 6:**
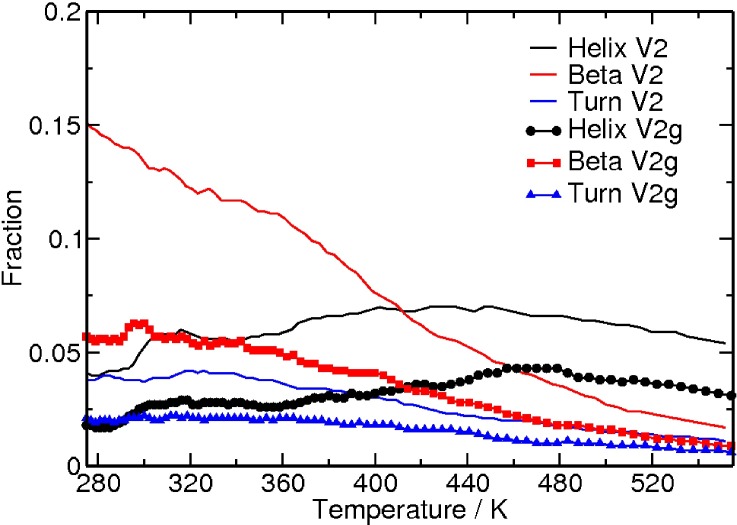
Fraction of residues that form helix, beta-strand or turn structure as a function of increasing temperature. Both glycosylated (symbols) and unglycosylated (no symbols) cases are shown.

An initial set of REMD simulations was carried out to examine how glycosylation at the conserved positions N156 and N160 affects the secondary structure propensities of the unstructured, flexible V1V2 peptide construct. This isolated peptide exists predominantly as a random coil, and the fraction of residues that form a helix, beta-strand, or turn structures as a function of temperature is plotted in **Fig 6**. While the fraction of residues in the glycosylated and unglycosylated peptides involved in beta-strand or turn structures decreases with increasing temperature, the fraction of residues that form a helix structure increases until around 440 K, followed by a slight decline. Importantly, a larger fraction of the residues are prone to form secondary structures other than random coil (helix, beta-strand, or turn structures) in the unglycosylated form compared to the glycosylated form (**Fig 6**). Thus, the overall probability of the V1V2 peptide to form folded secondary structures is reduced upon glycosylation.

### Glycosylation modifies the free energy landscape of the V1V2 peptide construct

**Fig 7:**
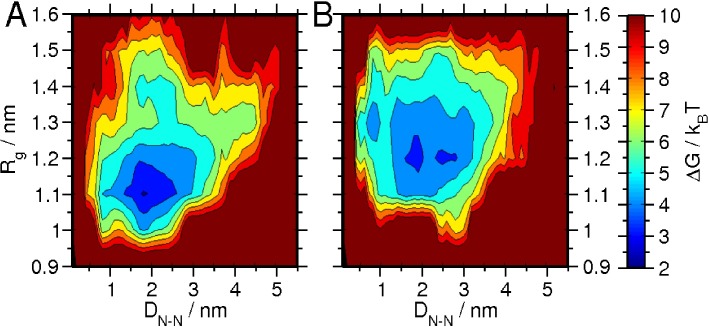
Influence of glycosylation on the free energy landscape of the peptide without (A) and with (B) glycosylation. Landscape is considered as a function of end-end distance and radius of gyration. The contour plots are in units of k_B_T, and the difference between neighboring lines is 1 k_B_T.

The free energy landscape of the V1V2 peptide in terms of end-end distance and radius of gyration is shown in **Fig 7**. In the absence of glycosylation, 43% of the configurations were enclosed within a single state (R_g_ = 1.1 nm, D_N-N_ = 1.8 nm) (**Fig 7A**). Upon glycosylation however, 20% of the total configurations populated two states: (R_g_ = 1.2 nm, D_N-N_ = 1.8 nm) and (R_g_ = 1.2 nm, D_N-N_ = 2.5 nm) (**Fig 7B**). These results suggest that the overall ensemble of the V1V2 peptide is more extended upon glycosylation. This is consistent with the reduction of secondary structural preference as seen in **Fig 6**. Such an effect can arise with increased entropy of a peptide backbone, disruption of intra- and inter-peptide interaction, and between peptide and glycan, each of which were subsequently investigated.

### Glycosylation affects the backbone flexibility of the Asn156 and Asn160 residues

**Fig 8:**
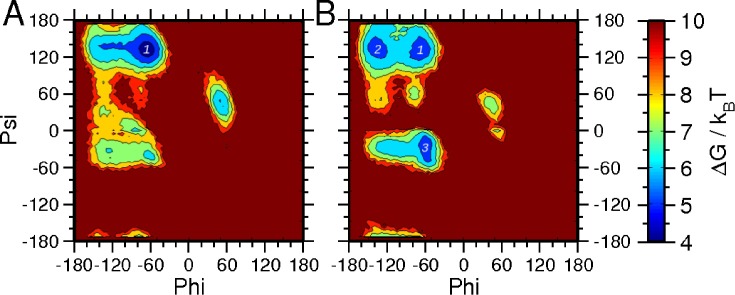
The backbone Ramachandran plot of the residue N156 for the two peptide systems without (A) and with (B) glycosylation is shown. The polyproline II (1), extended beta (2) and right-handed alpha-helical (3) regions are marked. The contour plots are in units of k_B_T, and the difference between neighboring lines is 1 k_B_T.

While N-linked glycosylation is often linked with global conformational effects, it is possible that the addition of carbohydrate could affect the N residue itself. A Ramachandran plot was therefore determined for the N156 backbone dihedral angles (**Fig 8)**. Without glycosylation at N156, the most sampled region shows characteristics shared with polyproline II motifs (-65°, 135°). It is followed by the right-handed and left-handed alpha-helix regions (**Fig 8A**). Glycosylation at N156 reduces the backbone sampling of polyproline II and left-handed alpha-helix regions and enhances the sampling of the extended beta-basin (-135°, 135°) and right-handed alpha-helix (-60°, -30°) regions (**Fig 8B**). To further quantify the differences in sampling, Shannon entropy was calculated with and without glycosylation at N156. The equation *S* = −*R* * *P*_*i*_ * *log*(*P*_*i*_) was used to calculate Shannon entropy, with *R* equal to the molar gas constant and *P*_*i*_ equal to the probability to sample each bin. This calculation gives entropic values of 63.01 kJ K^-1^ mol^-1^ n and 60.93 kJ K^-1^ mol^-1^ with and without glycosylation, respectively. The slightly higher Shannon entropy for N156 backbone sampling with glycosylation is consistent with varying bin size. Thus, by introducing a carbohydrate moiety, increased dimensionality is added to the peptide construct, resulting in higher backbone sampling and entropy.

### Glycosylation increases configurational entropy of the V1V2 peptide

Glycosylation of the V1V2 peptide at N156 and N160 could also alter the configurational entropy of the entire peptide. To investigate this, calculations were performed following the formulation of Schlitter[63]. S4 **Fig** shows the configurational entropy of the peptide backbone at 300K and 450K, averaged over a 250 ns simulation. Clearly, glycosylation increases the configurational entropy of the peptide, and is greatest at higher temperatures, consistent with the flexibility in secondary structure of the peptide. Interestingly, the inclusion of the glycans increases the entropy of the peptide backbone at higher and lower temperatures by ~100 and ~60 J mol^-1^ K^-1^, respectively. However, such an increase in entropy of the peptide backbone is somehow compensated by the enthalpic contribution of the glycan, as discussed below.

### Glycosylation disrupts intra- and inter-peptide hydrogen bonding interactions

**Fig 9:**
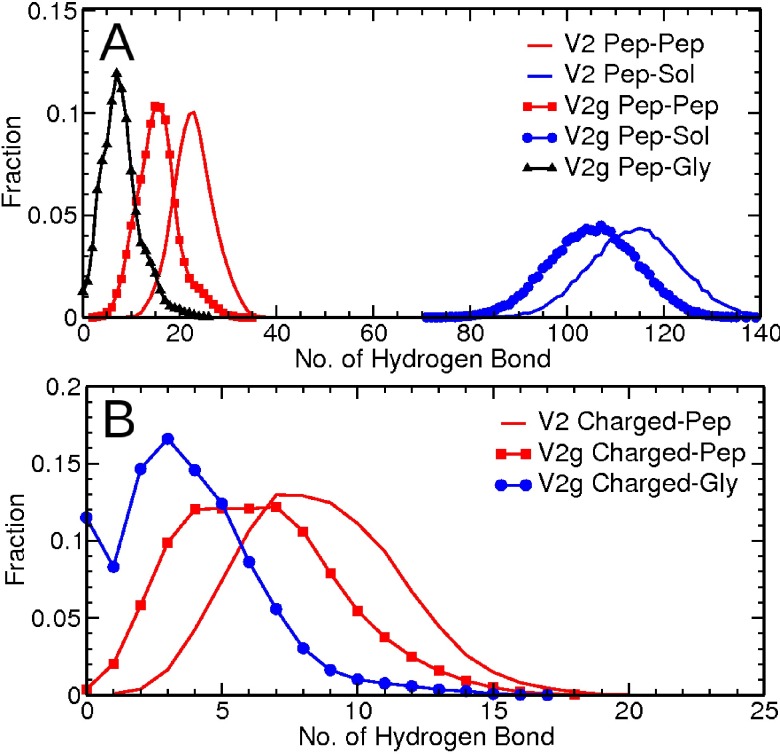
Fraction of configurations as a function of number of hydrogen bonds between different components of the system. (A) Total hydrogen bonding within the peptide, between peptide and solvent, and between glycan and peptide. (B) Hydrogen bonding between the glycans and charged residues. Pep stands for peptide, Sol for solvent water, Gly for glycan, and Charged for the charged residues of the peptide.

The effect of glycosylation on intermolecular interactions can be quantified in terms of hydrogen bonding within the peptide, between peptide and solvent, and between glycan and peptide. The total number of hydrogen bonds in these different types of interactions in terms of configuration is shown in **Fig 9**. Regardless of glycosylation, as expected, peptide-solvent hydrogen bonding dominates (**Fig 9A**). Overall, there are much more hydrogen bond interactions between the peptide and water molecules compared to intra-peptide hydrogen bond interactions. However, glycosylation does disrupt hydrogen bonding between peptide and solvent, and between peptides.

### Glycosylation increases the stability of peptide through enthalpic contributions

**Fig 10:**
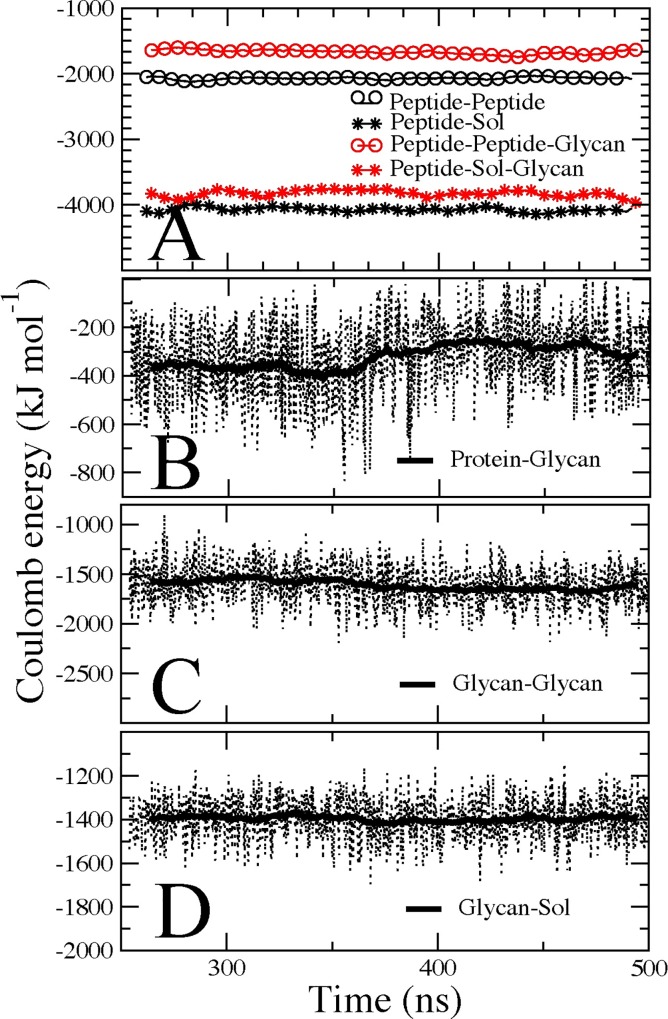
Electrostatic contribution from glycan to the stability of the peptide at 300K. Different contributions are depicted and averaged along the simulation time. **(A)** intra and inter-molecular interaction of the peptide, with or without the glycan. **(B,C,D)** Coulomb contributions after the addition of the glycan to the peptide.

As shown above, glycosylation of the V1V2 peptide disrupts the intra- and inter-molecular hydrogen bonding of the peptide. However, this effect could potentially be compensated by the hydrogen bond interactions that arise due to introduction of glycan. Additionally, the electrostatic nature of the glycan can lead to specific interactions with charged residues of the peptide. Therefore, the different coulombic contributions to the overall energetics were considered to capture any compensating electrostatic interactions introduced by glycosylation. Accordingly, even though several hydrogen bonding interactions involving charged residues are reduced by addition of the glycans, the reduction is compensated by de novo hydrogen bonding between the glycan and other polar residues (**Fig 9B**). **Fig 10A** shows the averaged coulombic contribution as a function of time for the interactions between the peptide, glycans, and solvent. The addition of the glycan clearly affects the intra-molecular interactions of the peptide, as well as the peptide interactions with water molecules. However, the presence of the glycans adds ~ 3000 kJ mol^-1^ to the stability of the peptide (**Fig 10**, sum of the energies from panels **C** and **D**). On average, most of the contribution comes from the interaction between the two glycans and the interaction of glycans with the solvent. This observation was also recorded at a higher temperature. Overall, these results suggest that the glycan itself contributes enthalpically to the stability of the peptide in solution.

### Glycan-glycan interactions are preferred over glycan-peptide interactions

**Fig 11:**
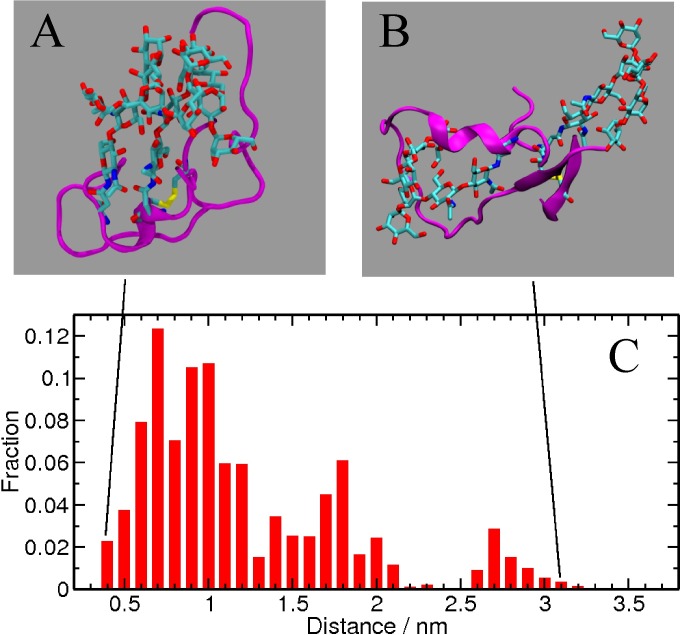
Fraction of configurations as a function of inter-glycan distance. Two representative configurations of shorter and longer inter-glycan distances are shown in (A) and (B). The peptide is shown as a purple ribbon, the glycans are in cyan and red stick representation, and the disulfide bond is shown in yellow. C) Cumulative fraction of the configurations as depicted in A and B. Clustering is based on glycan-glycan distance.

The interaction between the two glycans at N156 and N160 was characterized in terms of their contact distance. The inter-glycan distance is calculated as the distance between the two C1 atoms of the first mannose in each carbohydrate moiety. The fraction of configurations as a function of inter-glycan distance is plotted in **Fig 11**. Two snapshots of the glycopeptide are also shown, one with an inter-glycan distance of 0.4 nm (left panel) and the other with a distance of 3.1 nm (right panel). A short inter-glycan distance corresponds to the glycans interacting with each other. The inter-glycan distance distribution is highest between 0.75 nm and 1.0 nm, clearly demonstrating that the smaller inter-glycan distances are preferred. It is likely that antibodies that target that region can interrupt such glycan-glycan interactions. In the context of the flexible unstructured peptide construct considered in this study, spatially proximal glycans prefer to interact with each other.

### Glycosylation stabilizes the pre-existing conformation of the V1V2 peptide

**Fig 12:**
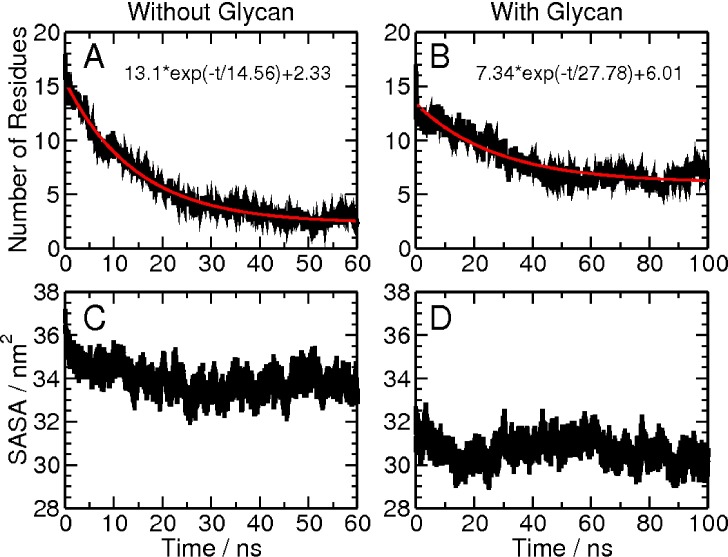
Thermal unfolding simulations. Panels **(A)** and **(B)** show the average number of residues in beta-strand as a function of time for the peptide alone and the glycosylated peptide; panels **(C)** and **(D)** show the average solvent accessible surface area (SASA) for the two systems as a function of time.

The V1V2 peptide construct utilized here is predominantly disordered when in solution. However, as mentioned above, it can adopt beta-strand or alpha-helical structures when bound to antibodies. To understand whether glycosylation can stabilize pre-formed beta-strand conformation, unfolding simulations were carried out at 450 K for the peptide construct starting from an initial beta-strand configuration similar to that in complex with the broadly neutralizing antibody PG9. Both the unglycosylated and glycosylated peptide systems were considered (see Methods section). The average number of residues in beta-strand as a function of time for the two systems is plotted in **Fig 12.** The beta-strand structures unfolded rapidly, disappearing within 60 ns of simulation for the unglycosylated peptide (**Fig 12A**). A fitted exponential curve provided a decay time constant of 14.56 ns. Only two residues remained in the beta-strand structure at the end of the simulation. In contrast, beta-strand structures unfolded at a much slower rate in the glycosylated system, and a significant amount of secondary structures remained at the end of the 100 ns simulation for the glycosylated peptide (**Fig 12B**). The decay time constant was 27.78 ns for the glycosylated peptide, and there were six residues remaining in the beta-strand at the end of the simulation. Thus, glycosylation of the V1V2 peptide retards the decay of preformed secondary structure by almost two-fold and preserves more residues with secondary structure. This potentially demonstrates the ability of glycosylation to stabilize the desired conformation of this region of V1V2 in terms of antibody recognition.

### Desolvation effects introduced by glycosylation

To explore whether some aspects of stabilization due to glycosylation could be attributed to shielding of solvent, the average surface accessible surface area (SASA) for the unglycosylated and glycosylated V1V2 peptide systems was determined, and is shown as a function of time in **Fig 12C** and **12D**, respectively. The SASA for the glycosylated peptide is less than that of the unglycosylated peptide, likely due to glycan shielding that may also contribute to the stabilization seen above. Previous studies have shown that desolvation of helix or beta-strand peptides stabilize the conformation by strengthening the intra-peptide hydrogen bond interactions[83, 84]. In addition, interactions between the glycan and the peptide residues can also provide stability as discussed above.

### Disulfide bonding promotes the formation of beta-strand structure

**Fig 13:**
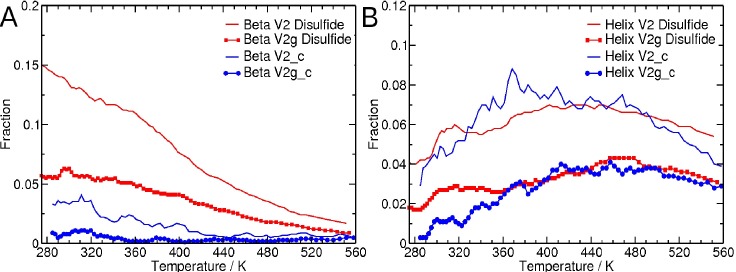
Secondary structure propensity of the glycosylated peptide. **(A)** Fraction of residues in the peptide that are in the beta-strand structure as a function of temperature for the glycosylated (V2g) and unglycosylated (V2) forms of CAP45 V1V2 peptide and the glycosylated (V2g_c) and unglycosylated (V2_cc) forms of ConC V2 peptide. **(B)** Fraction of residues in the peptide that are in the helix structure as a function of temperature for the glycosylated (V2g) and unglycosylated (V2) forms of CAP45 V1V2 peptide and the glycosylated (V2g_c) and unglycosylated (V2_cc) forms of ConC V2 peptide.

Next, we computationally investigated the possibility that disulfide bonds such as those found in V1V2 promote the formation of beta-strand structures in unstructured peptides by bringing two peptide regions close together. To investigate whether the disulfide bridge impacted the stabilizing effects of glycosylation on the V1V2 peptide, we considered a V2 loop fragment derived from a clade C consensus (ConC) sequence that did not contain the V1 fragment and the disulfide bond. The propensity to form secondary structures for the CAP45 V1V2 (with disulfide bond) and the ConC V2 peptide (without disulfide bond) is shown **in Fig 13.** In ConC V2 peptide, like CAP45 V1V2 peptide, glycosylation reduces the propensity for secondary structure formation. However, the fraction of residues that form a beta-strand structure is higher when there is a disulfide linkage, as seen in the glycosylated and unglycosylated forms of the V1V2 peptide (**Fig 13A**). Though, the helical content is lower for both peptides, in contrast, the fraction of residues that form a helix is reduced to the same level by glycosylation in the presence or absence of the disulfide bond (**Fig 13B**). Thus, our calculations tend to suggest that the propensity to form beta-strand structure is noticeably increased in the presence of the disulfide bridge.

### Effect of glycosylation on the V1V2 peptide region in the context of entire Env spike

To this point, we have presented computational results from studies performed on an isolated peptide fragment from the V1V2 region of gp120. An inevitable question is whether the effects of glycosylation described for this fragment are the same in the context of the entire Env trimer spike. To address this, we performed all-atom MD simulations of the Env spike using the BG505 SOSIP gp140 Env trimer in complex with broadly neutralizing antibody PGT122 [65]. A more extensive characterization of the global effect of glycosylation and its contributions to stability of the Env trimer are in progress elsewhere (manuscript in preparation).

**Fig 14:**
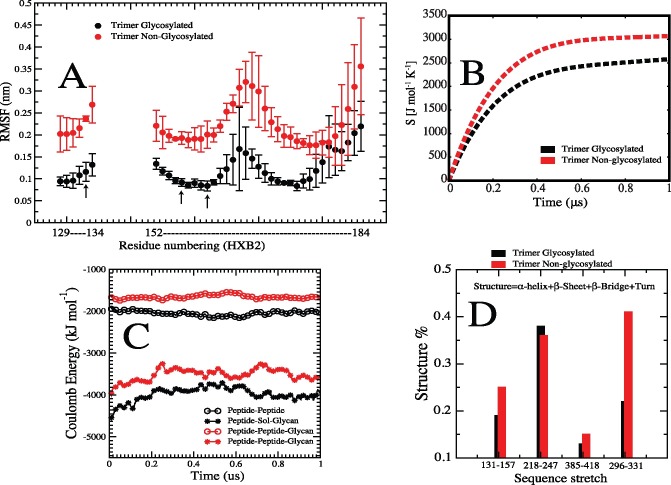
Effects of glycosylation on V1V2 peptide region in the context of the BG505 Env trimeric spike. **(A)** Root mean square fluctuation of the backbone atoms corresponding to residues 129–134 and 152–184 (HBX2 numbering) and computed for either the glycosylated (black line) and non-glycosylated (red line) protein. Error bars were estimated from calculation in each of the independent protomers. **(B)** Cumulative configurational entropy for the backbone atoms corresponding to the same residues as in panel A. Values were estimated by considering the total entropy from the three promoters. **(C)** Total interaction energy from the representative sequence as in B. The energy corresponds to the total value calculated among the three protomers and during 1us trajectory simulation. **(D)** Secondary structural percentage as computed from 1us MD simulations of the full Env spike. Four stretches were considered for the analysis, each featuring disulfide bonds and glycosylation sites. Computed secondary structure percentage for amino acid stretches that contain glycans adjacent to Cysteins (HXB2 numbering): 131–157 (analogous to the V1V2 peptide), 385–418 and 296–331. It further demonstrates, in the context of Env trimer, that glycosylation decreases the amount of alpha-helix, beta strands, bridge and turns in these regions.

We find that many of the physical trends of glycosylation are preserved in the context of Env spike for the same regions of gp120 considered in the V1/V2 peptide construct. From the all-atom simulations of the Env spike, **Fig 14A** shows the root mean square fluctuations (RMSF) of the backbone atoms encompassing the V1/V2 construct sequence. The RMSF observed for the non-glycosylated sequence are at least two times greater than the glycosylated counterpart, indicating a significant reduction in flexibility imposed by glycosylation. Furthermore, the accumulated configuration entropy over time for the same V1/V2 region (**Fig 14B**) shows reduced entropy upon glycosylation. We also calculated the different coulombic contributions to the overall energetics within the V1/V2 sequence stabilization. Again, as shown in **Fig 14C** for the different interactions between the local protein region representing the peptide construct, glycans, and solvent, the results in the context of Env spike are similar to those from the isolated construct (see **Fig 10 top panel for comparison**). Therefore, whether in the context of the Env trimeric spike or as an isolated fragment, the backbone mobility of this V1V2 peptide is restricted by glycosylation. Furthermore, the presence of glycan affects the local intra-molecular interactions among protein residues well as their interactions with water molecules.

### Importance of N-glycosylation sites adjacent to cysteine residues in the folding of the of the V1V2 domain

**Fig 15:**
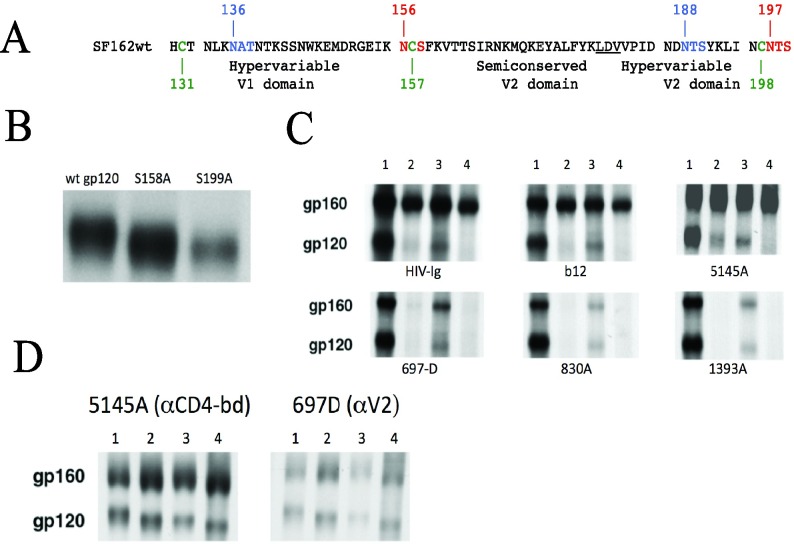
Effect of removing the glycosylation sites adjacent to Cys residues in V2 on processing of Env. **(A)** Sequence of wt SF162 Env protein. The two Cys-distal glycan motifs at positions 136 and 188 in the V1V2 hyper-variable region are shown in blue, while the two Cys-adjacent glycans at positions 156 and 197 in the semi-conserved V2 and C2 domains are indicated in red. Cys residues 131, 157, and 198 are indicated in green. **(B)** SDS-PAGE analysis of wt SF162 gp120 and gp120 containing mutated Cys-adjacent glycosylation sites at position 156 (S158A) and 197 (S199A). The increase in mobility is consistent with the loss of a glycan at these positions, confirming that these sites are in fact utilized. **(C)** Analysis of removing two Cys-adjacent glycan sites on intracellular processing of SF162 Env. Plasmids that encode wt and mutant SF162 Env proteins were transfected into 293T cells. Forty-eight hours post-transfection, the cells were radiolabeled with ^35^S-cysteine for 5 hours, and cells were lysed and immunoprecipitated with polyclonal HIV+ antiserum (HIVIG), or mAbs b12 and 5145A directed against conformational epitopes in the CD4-binding domain (top panels), or mAbs 697D, 830A and 1393A directed against conformational epitopes in the V1/V2 domain (bottom panels). Lane 1—wt Env, lane 2- S158A mutant, lane 3—S199A mutant, lane 4—S158A/S199A double mutant. **(D)** Analysis of removing two Cys-distal glycans on intracellular SF162 Env processing. Cells transfected with wt SF162 Env (lanes 1), T138A (V1) mutant (lane 2), S190A (V2) mutant (lane 3) and T138A/S190A double mutant were labeled for 5 hrs, and then cells were lysed and labeled Env proteins immunoprecipitated with mAbs recognizing conformational epitopes in the CD4-binding domain (5145A) or in the V1/V2 domain (697D). Precursor gp160 and processed gp120 bands are indicated in (C) and (D).

The studies described above indicate an important contribution of the highly conserved glycan at position at 156 adjacent to Cys157 in folding of the adjacent V2 peptide. The extreme conservation of this glycan in HIV-1 and among all primate lentiviruses (Cys196) also supports its role as an important element in the structural framework of the region (**S5 Fig**). These observations raise the question of whether this and other Cys-proximal glycans regulate the efficiency of processing and conformational folding in the context of the native Env protein. This was investigated by removing the 156 and 197 glycans and evaluating their effect on Env processing. To simplify this analysis, these studies were performed with SF162 Env, which conserves glycans 156 and 197 but lacks a glycan at position 160 (**Fig 15A**). For comparison, glycans in the same region that were not adjacent to the Cys residues, N136 and N188, were also mutated. The glycans at 156, 197, 136, and 188 were each eliminated by mutating the Ser or Thr residues in the corresponding motifs to Ala residues (**Fig 15A**).

Mutation of 156 and 197 glycosylation motifs resulted in a reduction in size of the corresponding gp120 proteins (**Fig 15B**). This was consistent with the loss of an N-linked glycan, and showed that both of these positions were glycosylated in the wild type (wt) Env protein. The effects of these mutations on Env folding were examined by comparing the intracellular forms of Env present after a 5 hr labeling period for the wt Env (lane 1), mutants containing the 156 or 197 mutation (lanes 2 and 3), and a 156/197 double mutant (lane 4) (**Fig 15C**). Immunoprecipitation performed with polyclonal HIVIG showed that the majority of the mutant Env protein remained in the unprocessed gp160 form. Processing to gp120 appeared to be impaired to a greater extent for the 156 glycan (lane 2) compared to the 197 glycan (lane 3), while processing was completely abrogated for the double mutation (lane 4).

Similarly impaired processing to gp120 was observed for samples immunoprecipitated with mAbs b12 and 5145A, which are specific for conformational epitopes in the CD4-binding domain (**Fig 15C**, upper panels). These antibodies recognized similar levels of the wt and mutant gPr160 precursors, indicating that these mutations did not affect the folding events required for the formation of the CD4-binding domain. However, recognition of gPr160 by mAbs to three conformational epitopes in the V2 domain was significantly reduced by mutation of the 197 glycan, and almost completely inhibited by the loss of the 156 glycan and the double mutation (**Fig 15C**, lower panels). Previous epitope mapping studies with mAb 830A indicated that this antibody recognizes a discontinuous conformational epitope that overlaps the α β -integrin binding site at positions 179–181. This epitope also involves other residues in both the V1 and V2 domains, and a crystal structure of a V1V2 scaffolded molecule complexed with 830A demonstrated that this epitope did not include any glycans [45], suggesting that the reduced recognition of the glycosilation mutants by the V2-specific antibodies was not due to direct mutation of these epitopes. A similar analysis of the 136 and 188 glycan mutants, which are not adjacent to the Cys residues, revealed that processing of the single and double mutant proteins was similar to the wt protein (**Fig 15D**). These results indicate that processing of gp160 to gp120 was significantly impaired by loss of the 156 and 197 glycans, but not by loss of the 136 and 188 glycans. Furthermore, the conformation of the V1/V2 domain was altered by removal of the two Cys-adjacent glycosylation sites, such that recognition by three conformationally-dependent V2-directed mAbs was significantly reduced.

## Discussion

Structural information about the effects of glycosylation on HIV-1 gp120, and in particular the hyper-variable domains, is still limited. This is due in part to the difficulties in obtaining crystal structures with glycans and hyper-variable domains intact. MD simulations can thus provide unique insight that enhances our understanding of the glycosylated gp120 structure. In this study, we used the enhanced sampling approach of replica exchange MD simulations to investigate the effects of glycosylation on a flexible unstructured region of the V1V2 domain that is of immunologic interest. We generated a peptide fragment containing portions of the V1 and V2 loops, including an antigenic region of gp120 that was recognized by antibodies generated by vaccination and during HIV-1 infection. The two regions were linked by a disulfide bond, and contained high mannose glycans at positions N156 and N160, which are targeted by a class of broadly neutralizing antibodies. These studies were undertaken to provide information about how to mimic salient structural features of V1V2 that could enhance its immunogenicity, as well as to understand the selective pressures that underlie strongly conserved features within a highly variable domain.

The addition of glycans to the V1V2 peptide caused a strong enthalpic compensation that resulted in two complementary effects on this disordered flexible fragment. First, glycosylation stabilized the pre-formed conformation of this peptide. Second, it reduced the propensity of the unstructured peptide to form secondary structures. Paradoxically, glycosylation destabilized this disordered V1V2 fragment by reducing its secondary structure propensities, while at the same time stabilizing it by preventing the peptide fragment from unfolding. It is possible that with glycosylation, the free energy of the unfolded state is lower due to the increase in entropic and enthalpic components. In addition, glycosylation also disrupts intra- and inter-peptide interactions that might be important to the folding process of the peptide. The large volume of a carbohydrate moiety could also impede the rearrangement of the V1V2 structure during its folding process. On the other hand, it is possible that the free energy of the folded structure is also lower with glycosylation due to introduction of strong interactions between glycan-glycan and glycan-solvent. Also, glycosylation reduces the solvent accessible surface area of the peptide, thereby shielding the unfolding process from the solvent.

Based on our results, we postulate that this destabilization of secondary structure is a generalized effect of the glycan attached to unstructured regions of proteins. At the same time, our results show that glycosylation can prevent a pre-formed beta-strand structure in the peptide from thermal unfolding; the unfolding process was much slower in the presence of glycosylation. One could envision that such a situation arises during oligomerization of gp120 monomers or when V1V2 is bound to an antibody.

Even though addition of glycans to the V1V2 peptide disrupted significant peptide-peptide and peptide-solvent interactions, a much larger favorable enthalpic contribution was obtained from the glycan-glycan and the glycan-solvent interactions. In fact, the large enthalpic contribution from solvation leads to better hydration of the peptide. This is reflected in the free energy landscape that favors an extended conformation of the peptide in the water solution. Additionally, a significant enthalpic contribution originates from glycan-glycan interactions in the case of glycosylation sites that are spatially proximal in the peptide, such as N156 and N160. Our studies show that if two glycans occur at spatially close sites in a flexible region of the protein, they will cluster together. There is an additional site, N130, often found next to a disulfide linkage in HIV Env (**Fig 1C**), although not in the CAP45 sequence that we used in this study; when it is present, it might also impact the conformation of both the peptide and the intact trimer as discussed below.

In this study, we characterized the changes in the energy landscape and thermodynamics of an isolated, disulfide bound V1V2 peptide fragment upon glycosylation. Such a characterization is critical for designing an immunogen construct involving glycosylation to ensure that the important conformational characteristics of the peptide are not significantly altered. It is also possible that the effect of glycosylation is more dramatic in a peptide construct when the scaffolding influence of the rest of protein is absent. In order to address this potential limitation, we performed extensive all-atom MD simulations of the entire Env spike and found that glycans exert similar effects on the V1V2 immunodominant region even when considered in the context of the whole Env gp120 trimer. Thus, backbone mobility is restricted when glycosylation is present in the context of the isolated peptide and the Env trimer, Furthermore, our simulations revealed that the presence of the glycan affects the local intra-molecular interactions among protein residues well as their interactions with water molecules.

All-atom MD simulations of the Env spike also demonstrated that additional glycans from the trimeric complex interact with the glycan moieties at positions 156 and 160. Preliminary results from the Env spike simulations show that the glycan at position 156 makes contact with other glycans within the same protomer, whereas the glycan at position 160 interacts with glycans from neighboring protomers at top of the spike (**S6 Fig**). Thus, it is likely that glycan interactions with 156 and 160 contribute to the stabilization of the V1/V2 immunodominant region and, more importantly, to the integrity of the trimer. Indeed, studies are currently underway to understand how glycan-glycan interactions contribute to the stability of the trimer.

Another study used simulations to investigate the effects of glycosylation on the mobility and conformation of the V3 domain in gp120 [85]. In that study, unglycosylated gp120 was compared with gp120 containing either a single or multiple proximal high mannose N-linked glycans. That study reported that glycans surrounding the disulfide bounded V3 domain modulate its dynamics and conformational properties. The glycans tended to constrain the movement of V3, and cause it to adopt a more narrow conformation than the non-glycosylated gp120 form. Interestingly, the glycans flanking the V3 domain are less well conserved across HIV-1 clades and circulating recombinant forms (CRFs) than those in V1V2. The N-linked glycan motif at N295, which is N-terminal to V3, is found less frequently in clades A and C compared to other group M clades and CRFs. At the C-terminal flank of V3, the N334 glycan addition site is highly conserved in CRF01_AE, but is more variable in other clades and CRFs, which tend to have higher frequencies of the N332 glycan addition site instead. Thus, the cysteine-bounded loops of gp120 are often flanked by glycans that are conserved to varying degrees, and therefore may be particularly susceptible to their influence.

Consistent with this concept is strong evidence that the placement of N-linked glycosylation sites adjacent to disulfide bonds is a highly conserved feature at the base of the V2 loop (and also modestly conserved at the base of V1, V3, and V4). Disulfide bonds are commonly found in proteins, but their effects on protein structure are still under investigation[10, 86–89]. Moreover, the effect of having a glycan juxtaposed to a disulfide bond has not been addressed from a structural perspective. In the peptide construct considered in this study, the glycan at 156 is adjacent to a disulfide bond. Past studies have shown that elimination of 156 and 160 glycans in the HIV-1 DH12 infectious molecular clone resulted in a loss of infectivity that was attributed to a defect in CD4 binding [90]. Furthermore, our studies demonstrated that removal of 156 and 160 from the HIV-1 SF162 Env impaired gp160 processing to gp120 and disrupted the conformation of V1V2. Mutation of V1V2 glycans that were not adjacent to Cys residues did not reproduce these effects. Thus, it is likely that Cys-adjacent glycans play an important role in HIV-1 Env processing, folding, and function.

We also evaluated a peptide region of V2, based on the consensus C sequence, that lacked the disulfide bond and the V1 fragment. By comparing the effects of the disulfide bond with or without glycosylation in the V1V2 and a second V2 peptide, we found that the residues adjacent to the disulfide bonded cysteine residues had a higher propensity to form beta-strand structure compared to other residues in the peptide (**S7 Fig**). However, addition of a glycan next to the disulfide diminished the propensity to form beta structures. In the context Env trimer, examination of other V1V2 and other disulfide regions with proximal glycans revealed that secondary structures are slightly diminished by the presence of glycosylation (**Fig 14D**). Taken together, these findings suggest that the preservation of the glycan next to the disulfide bond is most beneficial during folding or upon pre-forming a stable secondary conformation induced by binding to other proteins or antibodies.

Presumably, a major function of the Cys-adjacent glycans is to regulate the efficiency and specificity of disulfide bond formation. Intuitively, the large size of the glycans would limit degrees of rotation, which could regulate the orientation of the two peptide strands on either end of the disulfide. This may be particularly important in cases where there are proximal glycans to both partners of a disulfide bond, such as is observed for the Cys 131- Cys 157 disulfide bond that closes V1 and the disulfide bond at the base of the V3 loop. A somewhat under-appreciated consideration is that recombinant gp120 proteins possess considerable heterogeneity specifically in the V1V2 region [91], which could be influenced by the adjacent glycosylation. Consistent with this concept, mass spectroscopy studies have provided evidence for alternative disulfide pairing in the V1V2 region of the recombinant CON-S gp140 ΔCFI protein [91] and this may be influenced by glycosylation. There is also evidence that disulfide reorganization occurs after receptor binding and is mediated by membrane-associated protein-disulfide isomerases [92]. This could also be affected by proximal glycans. Thus, further studies are warranted to explore whether these effects are present only in lentiviral glycoproteins.

Here, we addressed the effect of glycosylation of a V1V2 peptide that generally exists in a disordered conformation in solution. From the standpoint of immunogen design, thermodynamics elucidated from the current study provide insightful strategies to stabilize the V1V2 peptide and drive it towards the formation of beta-strand structures that could be desirable for eliciting broadly neutralizing antibodies over those that recognize linear, non-neutralizing or strain-specific V1V2 epitopes. The RV144 human vaccine trial elicited cross-reactive but weakly neutralizing antibodies directed against epitopes in V2 that were inversely correlated with the rate of HIV-1 infection [93]. Structural studies indicated that the key region of V2 (residues 168–176) recognized by vaccine elicited non-neutralizing mAbs CH58 and CH59, and the V1V2-targeted broadly neutralizing mAb PG9, can exist in multiple conformations [46, 47]. PG9 appears to preferentially bind to a beta strand conformation, whereas CH58 and CH59 may recognize alternate forms [46, 47]. An additional key feature of V1V2 directed broadly neutralizing antibodies such as PG9 is their ability to bind to glycans at N156 and N160, in addition to the underlying peptide (17). Alam et al.[40] described V1V2 glycopeptide immunogens that bind with high affinity to mature V1V2 broadly neutralizing antibodies and their putative germlines, but with much lower affinity to the vaccine-elicited, strain-specific V2 antibodies [40]. Also, they elegantly showed the importance of disulfide bonds in their peptide constructs, which is consistent with our findings. Therefore, glycopeptide immunogens represent a viable strategy to elicit V1V2-directed broadly neutralizing antibodies, but the effects of glycan-proximal disulfide bonds will also need to be considered. The region that intervenes between the V2 ‘epitope’ region and the Cys involved in V2 loop closure is a hyper-variable segment. Thus, peptides and scaffolds that encompass the end of the V2 loop and include the conserved glycosylation sites at the base of the V1V2 region, such as those described in[93], may better mimic the structure found in a native Env trimer. However, this region also spans sequences that are unique and highly distinctive between every isolate, a balance to consider in immunogen and reagent design.

Finally, one aspect that was not considered in the current study is the heterogeneity of carbohydrate forms at glycan sites, in particular at N156 and N160. A recent study using BG505 trimer protein demonstrated that N156 and N160 participate in a glycan ring at the trimer apex [52]. Both glycans were found to be predominantly of the oligomannose type, consisting of varying proportions Man₅_-9_GlcNAc₂. The authors proposed that glycan processing at N156 and N160 is likely to be constrained by inter-protomer contacts, resulting in minimal processing, although N160 tends to be more heterogenous than N156 [52]. Likewise, a study using a clade G SOSIP trimer demonstrated that the trimer apex is a region of glycan crowding, with less processing than glycans occurring in more dispersed regions [53]. The results of these trimer-based studies stand in contrast with that of Amin et al.[48], which evaluated cyclic V1V2 peptides that contain glycans attached at N156 (or N173 which substitutes for N156 in some isolates) and N160. Interestingly, the N-glycans at N156 and N173 have been shown to be spatially equivalent in terms of antibody recognition. The authors found that Man₅GlcNAc₂ glycan was required at N160 for recognition by broadly neutralizing antibodies PG9 and PG16. Furthermore, a sialylated N-glycan at the secondary site (N156 or N173) was also necessary for antibody binding to the glycopeptide. However, in the context of the BG505 trimer, PG9 binds regardless of whether the protein was produced in the presence or absence of glycan processing, supporting that these glycans are likely to be composed mainly of oligomannose forms in the native envelope trimer [94]. Taken together, these studies suggest that a better understanding of the structural features and immunogenicity of V1V2, and the conformational forms recognized by broadly neutralizing antibodies, could lead to the development of novel glycopeptide immunogens.

## Conclusion

A major difficulty in the pursuit of incorporating V1V2 epitopes into HIV vaccine design is the structural heterogeneity and variable glycosylation of this immunogenic region. Limited knowledge of how glycosylation and disulfide bonds affect the conformation and dynamics of short intrinsically disordered peptides complicates the design of immunogenic peptides. Thus, the development of strategies to define and exploit optimal configurations of V1V2 epitopes is important. Here, we used extensive replica exchange and conventional MD simulations to characterize the effects of glycosylation on the free energy landscape of a disulfide bound V1V2 peptide and dissect the enthalpic and entropic components upon addition of a glycan. Our analyses demonstrated that glycosylation stabilizes the pre-existing conformation of this peptide, and reduces its propensity to form other secondary structures. However, glycosylation also stabilizes the V1V2 peptide against thermal unfolding, and exhibits specific effects in relation to the adjacent disulfide linkage. These complementary effects originate from a combination of multiple factors, including the observation that having a disulfide bond adjacent to the glycan sites further promotes the formation of beta-strand structure in this peptide. Glycosylation and disulfide linkage are therefore likely important components that contribute to the immunogenecity of this region of V1V2, and will influence whether the appropriate conformation is adopted. The observation that HIV-1 is under strong selective pressure to conserve glycans adjacent to disulfide bonds could perhaps be exploited in the design of immunogens.

## Supporting Information

S1 Fig The HXB2 reference strain gp120 sequence was used to illustrate the association between glycosylation sites and the 8 Cysteines that form disulfide bonds at the base of the variable loops V1-V4. Of the 23 Cys residues in HXB2, 8 form the base of a variable loop, and 5 of the 8 have an adjacent N linked glycosylation site. In contrast, 0 of the 15 Cys residues that do not occur at the base of a variable loop have an adjacent N linked glycosylation site (Fisher’s exact, p = 0.0016).
(EPS)

S2 Fig Conservation of N-linked glycosylation sites N156 and N160 across 4633 HIV-1 group M sequences arranged by clade. Green indicates the fraction of sequences that contain both N156 and N160; red indicates that N156 is present; purple indicates that N160 is present, and blue indicates the fraction of sequences that lacks both sites. N160 tends to be absent more frequently in the B subtype, while N156 is absent more frequently in the D subtype.
(EPS)

S3 Fig Net charge distribution of the variable regions. Using the same input data as Fig 1, here we show that there is a great deal of charge variability in all of the variable regions. The V3 loop and V2 epitope region, despite being conserved in terms of length and number of glycosylation sites (Fig 1), both show a great deal of variation in net charge, comparable the level of diversity found in hypervariable regions. Net charge is calculated as the sum of positive and negative charges, where E and D are assigned -1, and K, D, and H are assigned +1. The V2 epitope region, V3 and V2 tend to be positively charged, V1, V4, V5, negatively charged.
(EPS)

S4 Fig Configurational entropy of the peptide backbone. The estimated entropy was obtained using 1200 frames of simulations at 300K (lines) and 450K (circles) from a total 250 ns trajectory, for both the single peptide (black) and after glycosylation (red). The small inset shows a close-up for higher resolution.
(EPS)

S5 Fig Conservation of N-linked proximal glycosylation sites between 72 HIV-2 and non-human primate representative lentiviral sequences from the HIV database. The V2 and V3 C-terminal proximal N-linked glycosylation sites at C196 and C331 are particularly well conserved.
(EPS)

S6 Fig Glycan-glycan interactions in the context of gp120 trimer of Env spike. Key glycan contacts in the V1V2 region are provided based on 1 us all-atom MD simulations of the fully glycosylated Env spike. The distances between the center of mass (COM) of glycans are shown. Distances are computed for inter-protomeric contacts (A) between glycans 133–156 (blue and red respectively) and a snapshot of the ensemble configuration is shown in the circled inset. Intra-protomeric contacts are shown for glycans located in position 160 (B) as well as for the pairs 185–156, 180–156 and 197–156 (C). Each protomer is denoted by a number from 1 to 3.
(EPS)

S7 Fig Fraction of each residue in the V2 and V2g systems that form beta-strand structures. The two blue-labeled “N” residues are the two glycosylated residues and the two red-labeled “C” residues are the two residues that are disulfide bonded.
(EPS)
